# Supplementary material for: ECT2 peptide sequences outside the YTH domain regulate its m6A-RNA binding
Source: RNA Biol. 2024 Sep 12;21(1):1–13. doi: 10.1080/15476286.2024.2399914 (PMC11404569; doi:10.1080/15476286.2024.2399914)
Supplement: Supplemental Material [file KRNB_A_2399914_SM6997.zip › Seigneurin Berny et al_revised Supplemental Figures.pdf]

Supplemental Figure 1

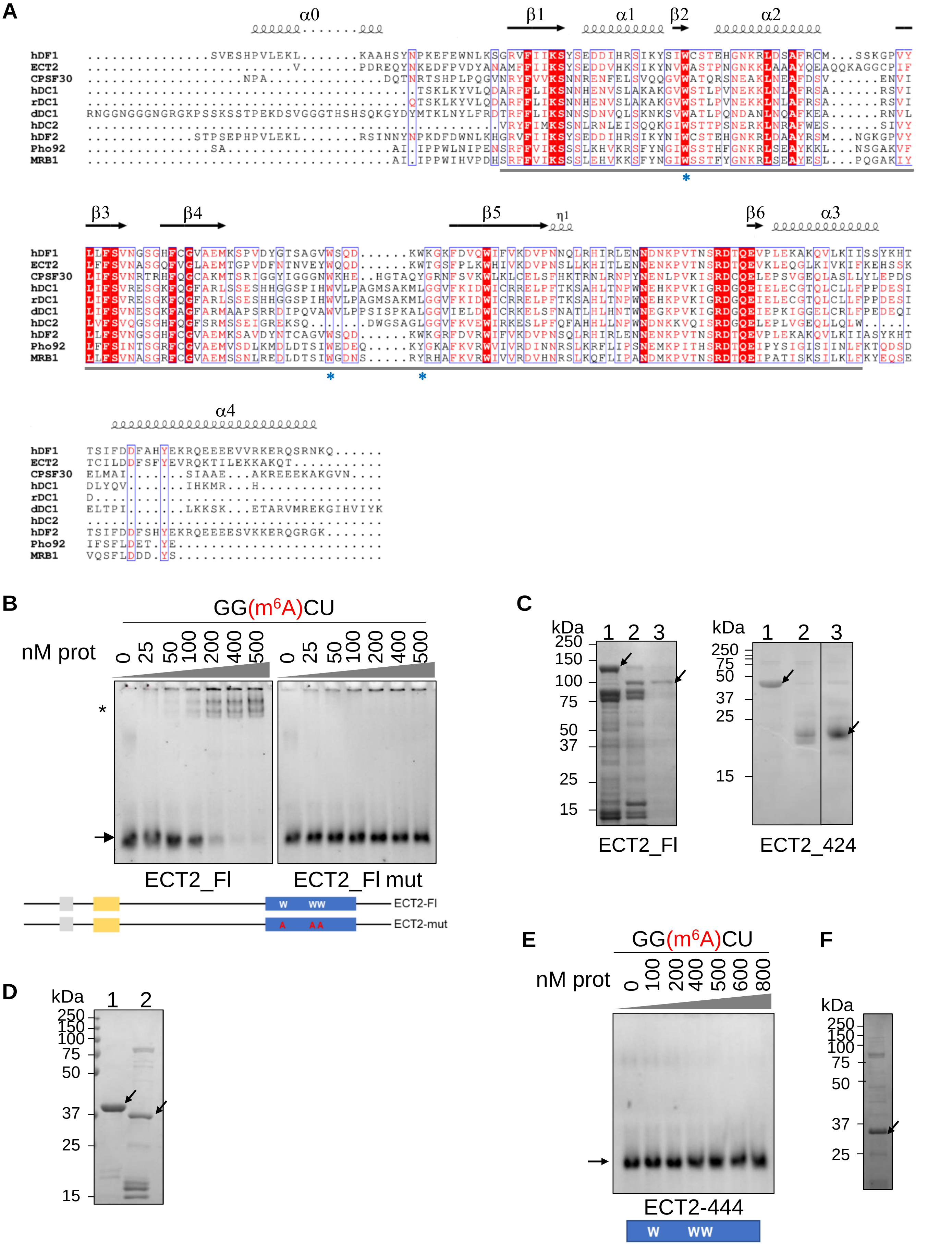

Supplemental Figure 1. YTH domain of ECT2 does not form a stable complex with m^6A-RNA *in vitro.*

A. Sequence alignment of extended YTH domains using ESPript 3.0 software [31]. The alignment was constructed with the sequences of the YTH containing regions used for the biochemical characterization as previously reported in the literature (EMSA, ITC and/or 3D structures) of human YTHDF1 (hDF1) aa 361-559, Arabidopsis CPSF30-L aa 220-400, human YTHDC1 (hDC1) aa 345-509, rat YTHDC1 (rDC1) aa 347-502, Drosophila YTHDC1 (dDC1) aa 204-423, human YTHDC2 (hDC2) aa 1288-1418, human YTHDF2 (hDF2) aa 380-579, *S. cerevisiae* Pho92p aa 141-306 and *Z. rouxii* Mrb1p aa 166-329. For Arabidopsis ECT2 the sequence from aa 424 to 610 (ECT2-424) was used. The structural elements of hYTHDF1 (PBD 4RCI) are reported above. The core YTH domain as defined in Uniprot is underlined in grey, the blue stars show the position of the amino acids forming the aromatic m^6A binding cage.

B. EMSA gels showing the binding capacity to the m^6A-RNA probe I of ECT2-Fl and a mutated version in which the three tryptophans required for m^6A binding were mutated into alanines (ECT2-Fl mut). Proteins are tagged with an N-terminal His-SUMO tag. After loading onto native acrylamide gels, the detection of free and bound probes was done using ChemiDocMP System (BioRad). The arrows indicate the free probe, the star the probe/protein complex.

C. Coomassie stained-gels showing the His-SUMO tagged ECT2-Fl and ECT2-424 proteins before and after TEV cleavage. 1: Ni-NTA purified His-SUMO tagged proteins, 2: His-SUMO tagged proteins incubated with His-tagged TEV, 3: purified recombinant proteins without tag after the second Ni-NTA purification.

D. Coomassie stained-gel of purified His-SUMO tagged extended YTH domains of CPSF30-L (aa 220-400, lane 1) and of hDC1 (aa 345-509, lane 2). In C & D, the arrows indicate the recombinant proteins.

E. EMSA gel performed with various concentrations of the purified His-SUMO tagged ECT2-444 construct (core YTH domain, aa 444-580) (from 0 to 800 nM) in presence of 10 nM of methylated 42 nt RNA probe I. The arrows indicate the free probe.

F. Coomassie stained-gel showing the His-SUMO tagged ECT2-444 protein. The arrow indicates the recombinant protein.

Supplemental Figure 2

A

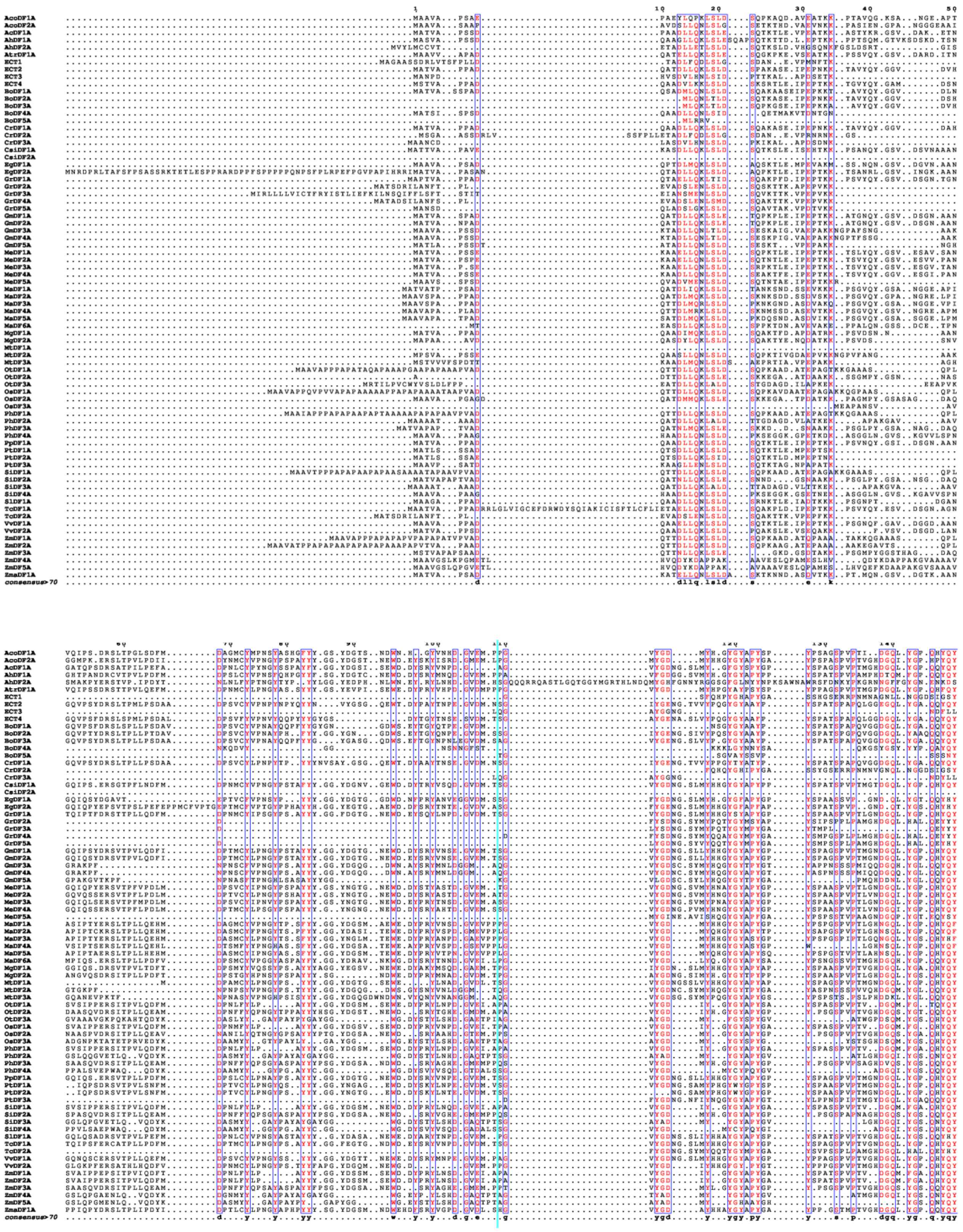

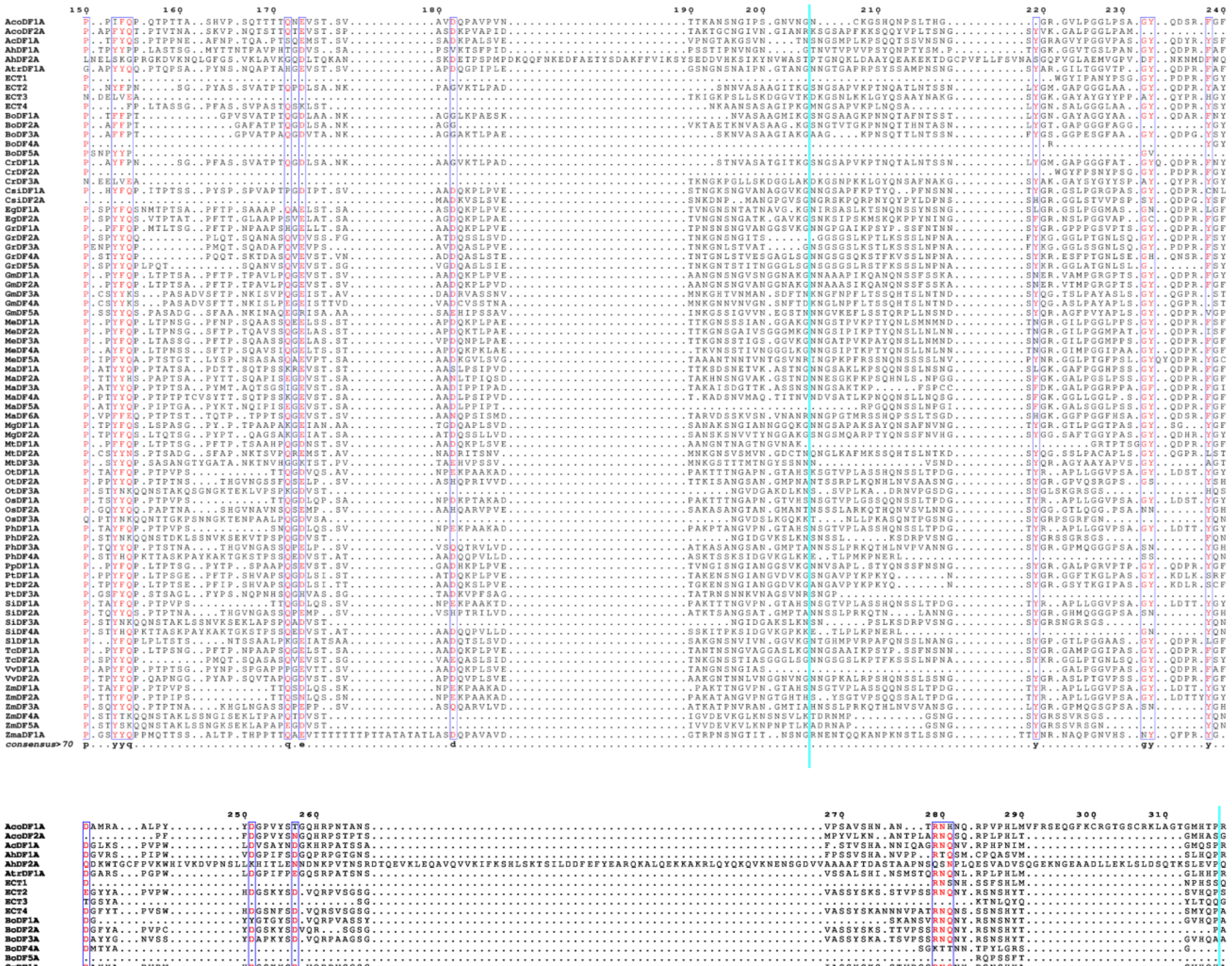

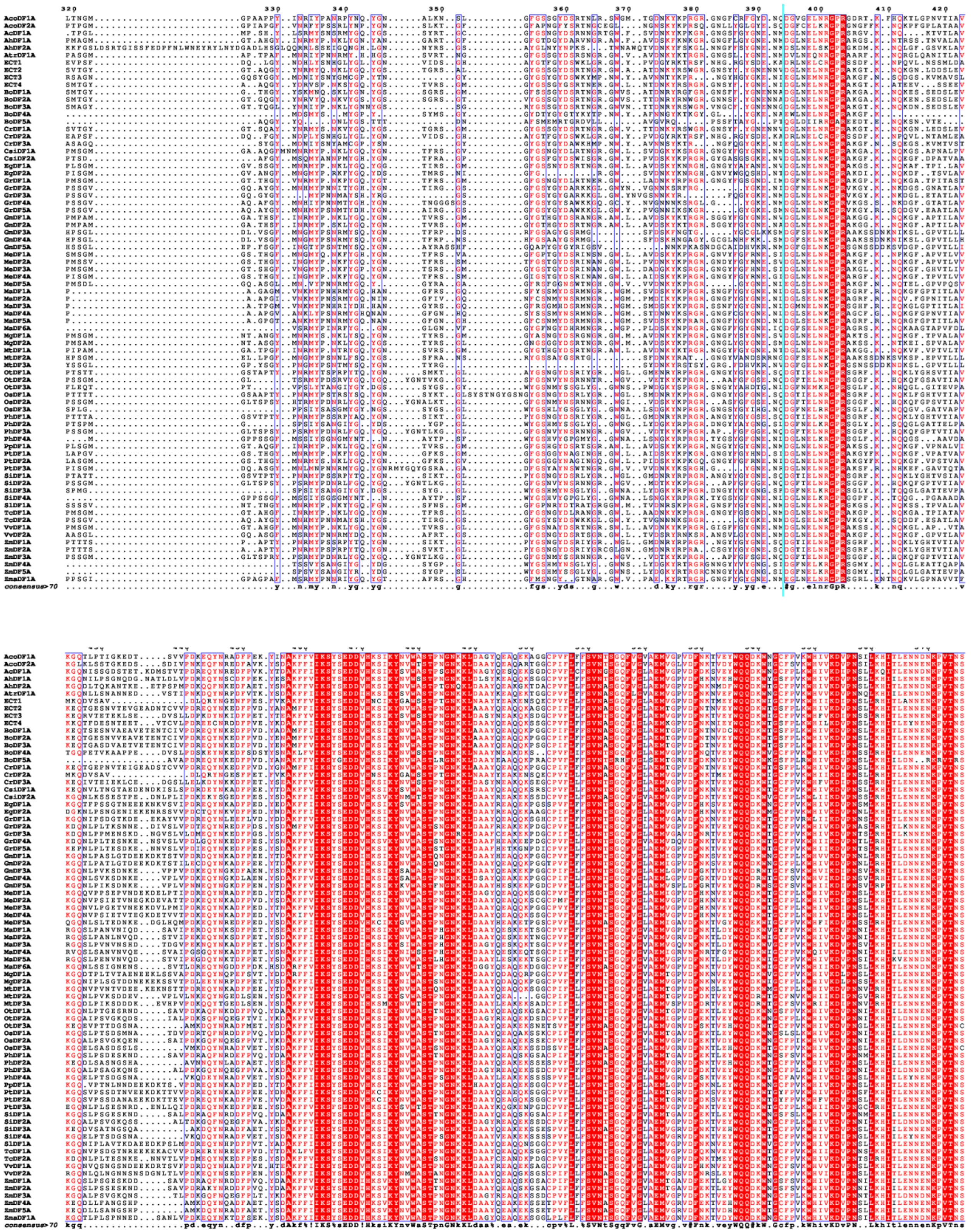

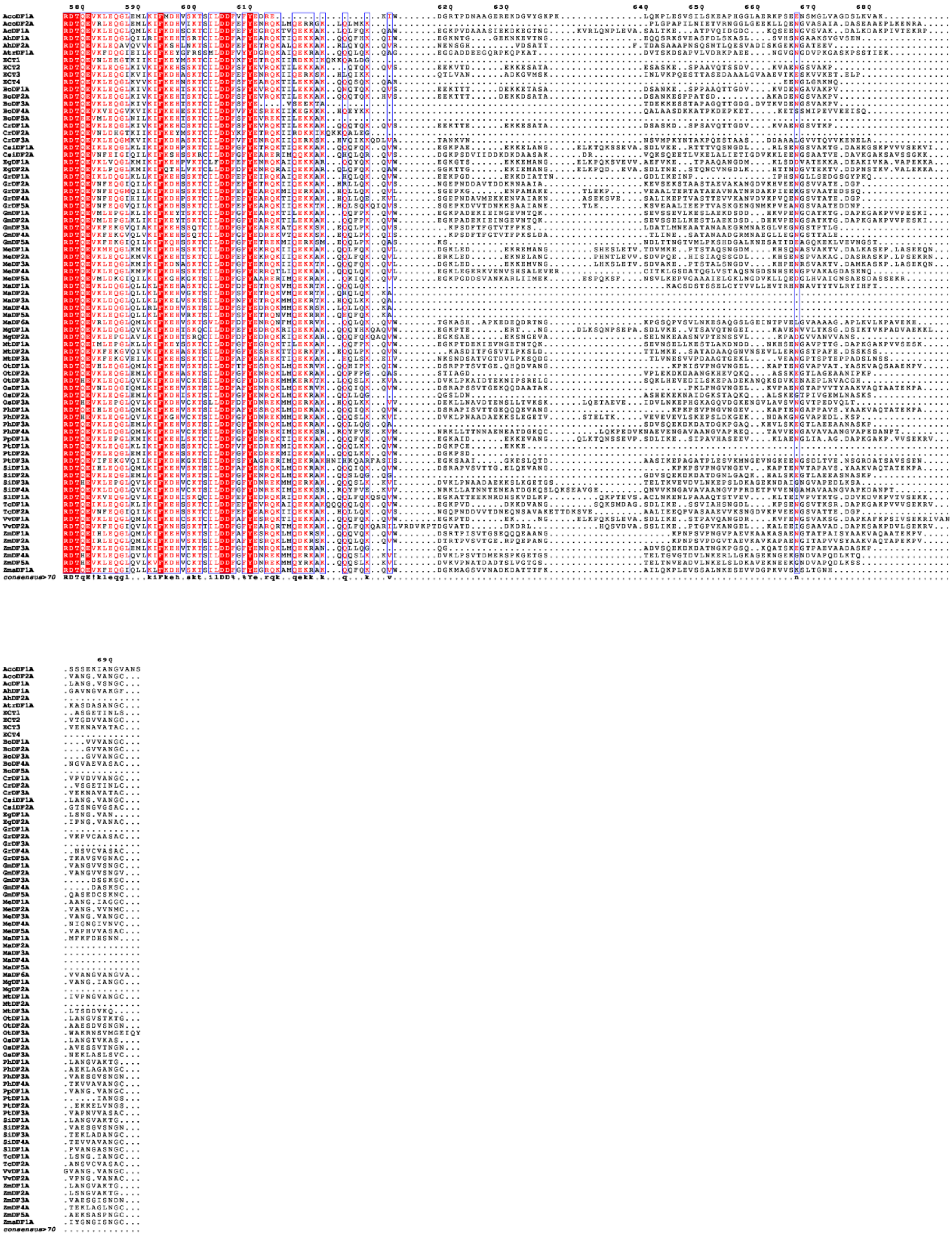

Supplemental Figure 2

B

MATVAPPADQATDLLQKLSLDSPAKASEIPEPNKKTAVYQYGGVDVHGQVPSYDRSLTPMLPSDAADPSVC
YVPNPYNPYQYYNVYGSGQEWTDYPAYTNPEGVDMNSGIYGENGTVVYPQGYGYAAYPYSPATSPAPQLGG
EGQLYGAQQYQYPNYFPNSGPYASSVATPTQPDLSANKPAGVKTLPADSNNVASAAGITKGSNGSAPVKPT
NQATLNTSSNLYGMGAPGGGLAAGYQDPRYAYEGYYAPVPWHDGSKYSDVQRPVSGSGVASSYSKSSTVPS
SRNQNYRSNSHYTSVHQPSSVTGYGTAQGYYNRMYQNKLYGQYGSTGRSALGYGSSGYDSRTNGRGWAATD
NKYRSWGRGNSYYYGNENNVDGLNELNRGPRAKGTKNQKGNLDDSLEVKEQTGESNVTEVGEADNTCVVPD
REQYNKEDFPVDYANAMFFIIKSYSEDDVHKSIKYNVWASTPNGNKKLAAAYQEAQQKAGGCPIFLFFSVN
ASGQFVGLAEMTGPVDFNTNVEYWQQDKWTGSFPLKWHIVKDVPNSLLKHITLENNENKPVTNSRDTQEVK
LEQGLKIVKIFKEHSSKTCILDDFSFYEVRQKTILEKKAKQTQKQVSEEKVTDEKKESATAESASKESPAA
VQTSSDVKVAENGSVAKPVTGDVVANGC-

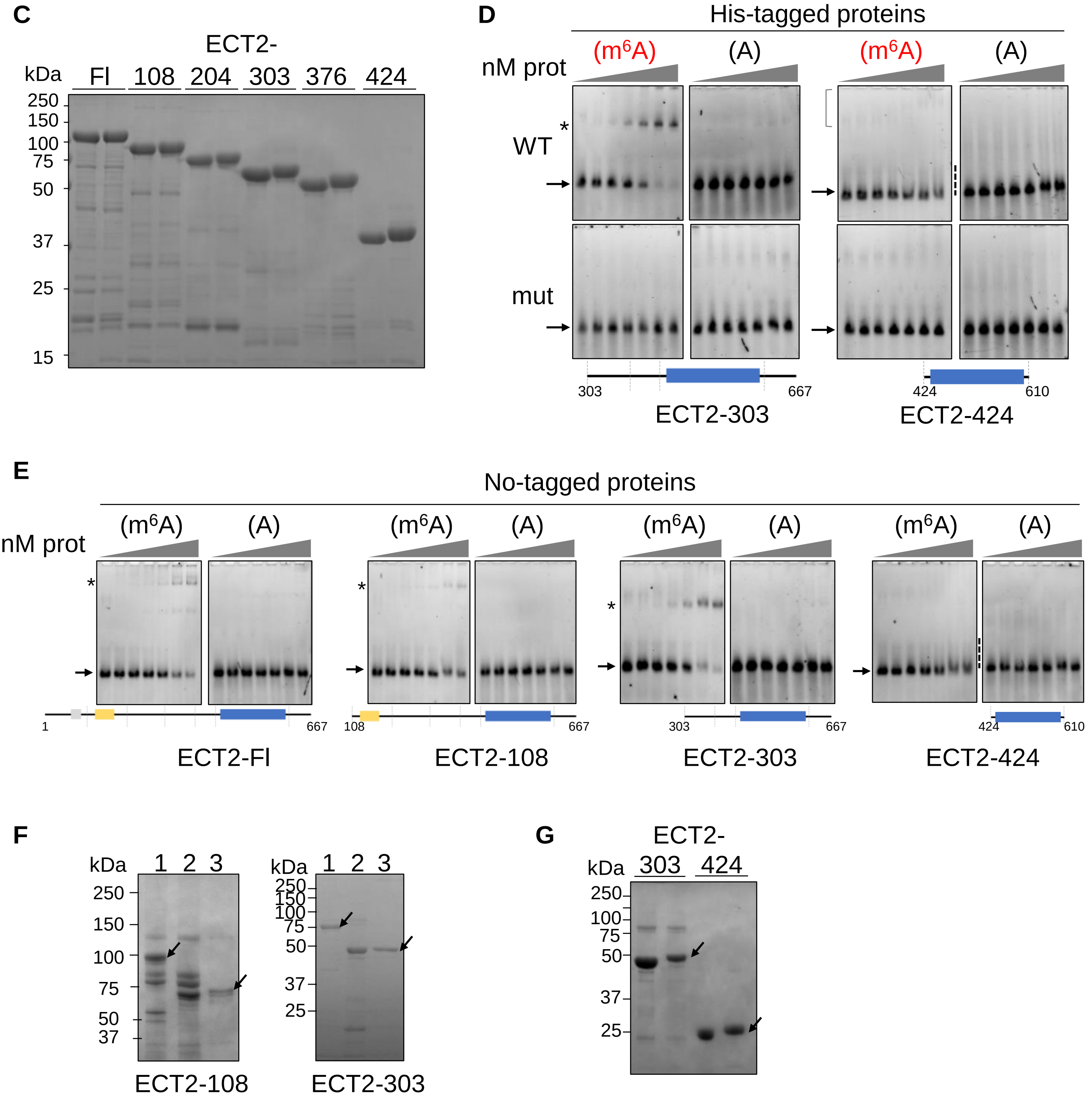

Supplemental Figure 2. ECT2 carries additional domains that modulate its binding to m^6A-RNA.

A. Sequence alignment of plant YTHDFA proteins done using the ESPript 3.0 software. The consensus residues are shown at the bottom (in bold). The sequences used for the alignment are listed in the Suppl. Table S3. The vertical blue bars correspond to the position of the truncated forms of ECT2.

B. Amino-acid sequence of ECT2 showing the conserved regions and residues shared amongst angiosperm YTHDFA proteins. Conserved residues are in bold, the SLiM domain [20] in grey, the YPQ-rich region [5] in yellow. In addition, are shown other domains recently described: the N3.2 (that comprises the SLiM) and N8 domains essential for ECT2 functions in leaf emergence [20] underlined in orange, and the N5 domain involved in antiviral defense underlined in green [18]. The YTH domain as determined in Uniprot is underlined in red, and the three tryptophans involved in the m^6A binding in red. The vertical blue bars mark the positions of the truncated forms of ECT2.

C. Coomassie staining of gel showing the recombinant His-SUMO tagged proteins. For each construct (ECT2-Fl, -108, -204, -303, -376, -424), a wild-type (left) and a mutant version (right), with the triple Trp to Ala substitution that inactivates the m^6A-pocket of the YTH domain, were produced.

D. EMSA gels performed with the proteins ECT2-303 and ECT2-424 tagged with 6 N-terminal Histidines. Protein concentration range was from 0 to 500 nM in the presence of 10 nM of methylated (m^6A) or non-methylated (A) RNA probes (probe I as in Figures 1 and 2).

E. EMSA gels performed with purified proteins after cleavage of the His-SUMO tag. For each assay, purified proteins (from 0 to 500 nM) were incubated with 10 nM probe I. For D & E, the free and bound RNA probes were visualized after loading of the reaction mixture onto native acrylamide gels and the probe fluorescent signal detected using ChemiDocMP System (BioRad). A schematic representation of all the proteins analyzed is shown under the corresponding gels. In D & E, the arrows indicate the free probe, the stars the probe/protein complexes, the dashed line, the smear in case of unstable complex. Non-specific signal is shown by the square bracket (for ECT2-424, in D).

F. Coomassie stained-gels showing the His-SUMO tagged ECT2-108 and ECT2-303 proteins before and after TEV cleavage. 1: Ni-NTA purified His-SUMO tagged proteins, 2: His-SUMO tagged proteins incubated with His-tagged TEV, 3: purified recombinant proteins without tag after the second Ni-NTA purification. The arrows indicate the recombinant proteins.

G. Coomassie stained-gels showing the 6His-tagged ECT2-303 and ECT2-424. Each protein was produced with a WT (left) or a mutated YTH domain (right). The arrows indicate the recombinant proteins.

Supplemental Figure 3

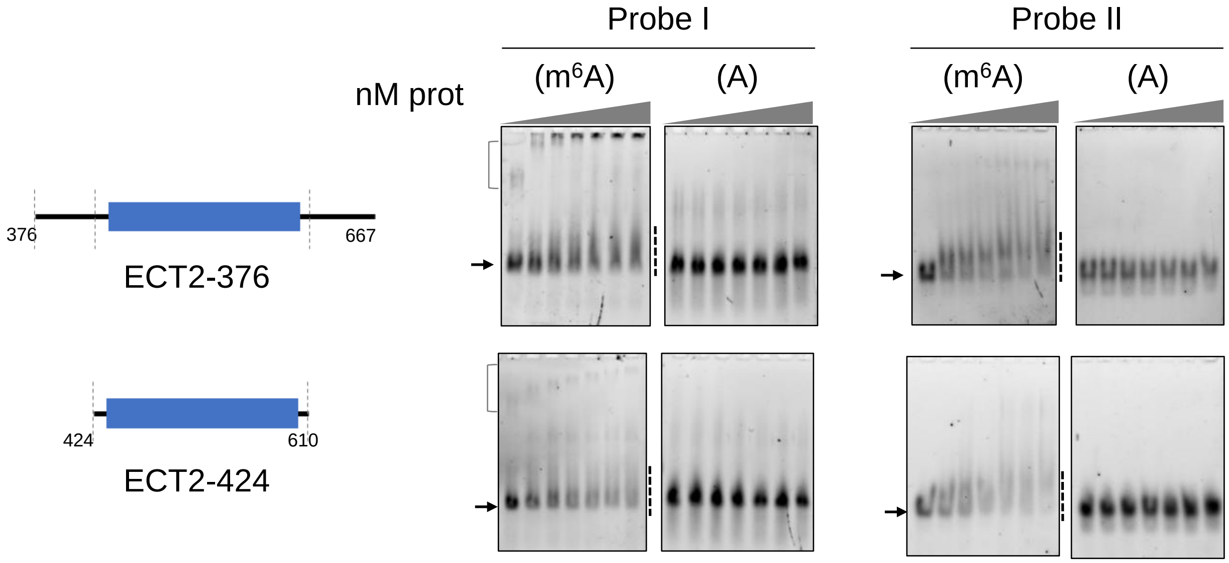

Supplemental Figure 3. Binding of ECT2 to m⁶A requires RNA sequences distant from the DRACH motif.
Analysis of the binding capacity of the His-SUMO tagged proteins ECT2-376 and ECT2-424. Each purified protein (from 0 to 500 nM) was incubated with 10 nM of methylated (m^6A) or non-methylated (A) probes. The reaction samples were loaded onto native acrylamide gels and the fluorescent signal of the probes visualized using a ChemiDocMP device (Biorad). A scheme of the proteins is shown on the left panel. The arrows indicate the free probe, and the dashed line, the smear observed when unstable complex. Non-specific signal is shown by the square bracket.

Supplemental Figure 4

A

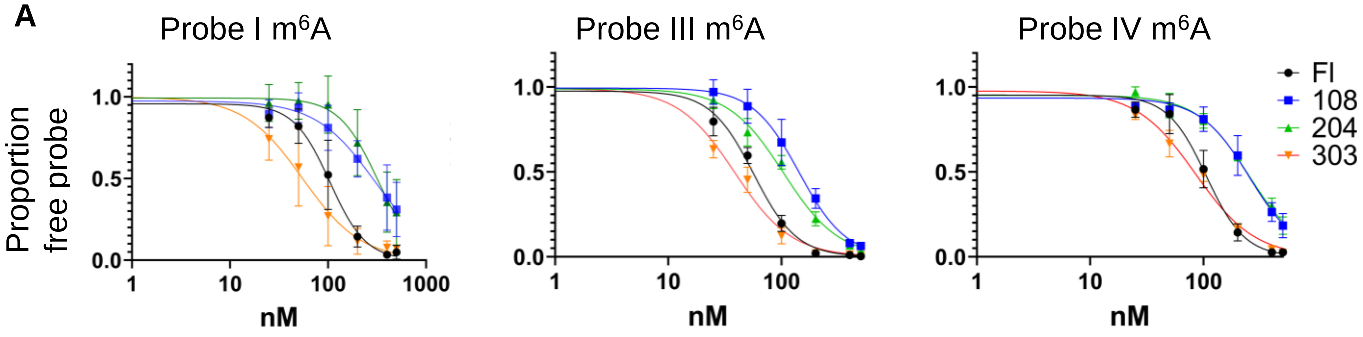


```
Probe I m6A:   5′-FAM-AUGGGCCGUUCAUCUGCUAAAAGG(m6A)CUGCUUUUGGGGCUU*G*U-3′
Probe III m6A: 5′-FAM-AAGGGCCGAACAACAGCAAAAAGG(m6A)CUGCAAAAGGGGCAA*G*A-3′
Probe IV m6A:  5′-FAM-AUGUGUCUUUCAUGUACUAAAAGG(m6A)CUGCUCUUGUGUCUU*G*U-3′
```

B

Kd (nM)	Probe I m6A	Probe III m6A	Probe IV m6A
ECT2 FI	105 +/- 8	57 +/- 3	106 +/- 7
ECT2-108	296 +/- 43	143 +/- 18	255 +/- 18
ECT2-204	318 +/- 35	104 +/- 6	253 +/- 15
ECT2-303	55 +/- 7	39 +/- 2	85 +/- 5

C

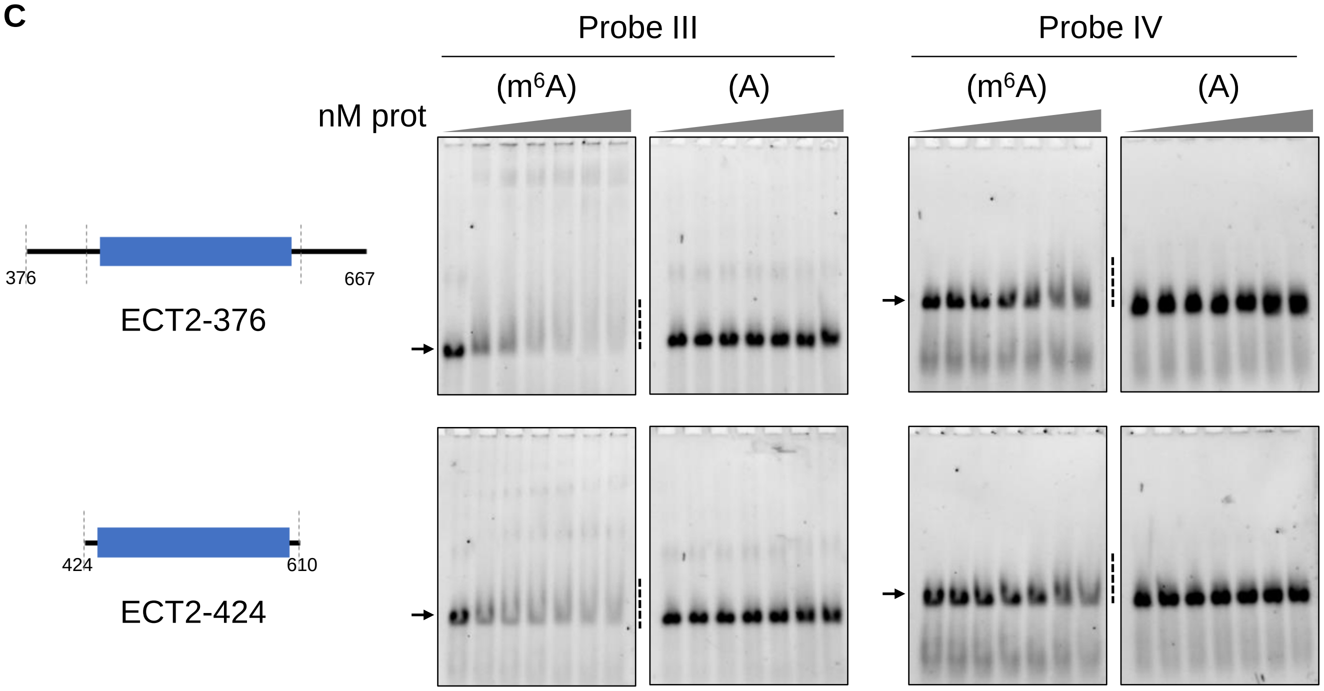

Supplemental Figure 4

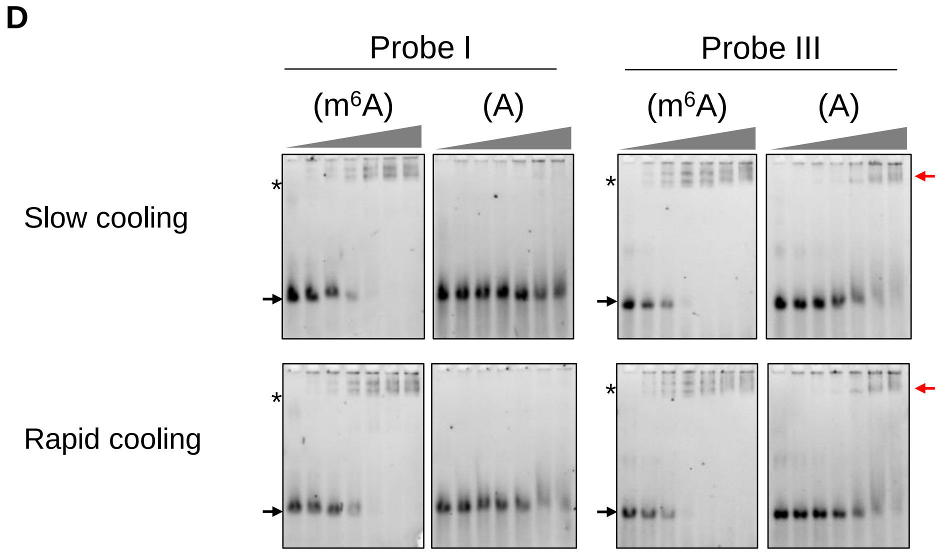

Supplemental Figure 4. The RNA sequence around the DRACH motif is important for the efficiency of ECT2 binding.

A. Quantification of the signals of the m^{6}A free probes obtained from EMSA displayed in Figure 4B. Graphs show the mean and standard deviation of at least three independent experiments. The curve fittings were obtained using the PRISM software. Each graph allows to compare the curve fittings obtained for the full-length and truncated forms of ECT2 with one methylated RNA probe (I, III or IV). The sequences of different probes are shown under the graphs.

B. From the curve fittings shown in A, the apparent Kd of each purified protein for each methylated probe were obtained using the PRISM software.

C. Analysis of the binding capacity of the His-SUMO tagged proteins ECT2-376 and ECT2-424. Each purified protein (from 0 to 500 nM) was incubated with 10 nM of methylated (m^{6}A) or non-methylated (A) probes III or IV. Scheme of the truncated proteins are shown on the left panel. The arrows indicate the free probe, and the dashed line, the smear observed when unstable complex.

D. EMSA gels obtained with His-SUMO tagged full-length ECT2 protein. In each assay, 10 nM of methylated (m^{6}A) or non-methylated (A) probes were incubated with increasing concentrations of purified proteins (from 0 to 500 nM). RNA probes I and III were used, after denaturation they were either slowly cooled down to room temperature (upper part) or rapidly on ice (lower part). The arrows indicate the free probe, and the stars the probe/protein complexes. The black squares indicate the probe/protein complexes detected with the non-methylated probe III.

Supplemental Figure 5

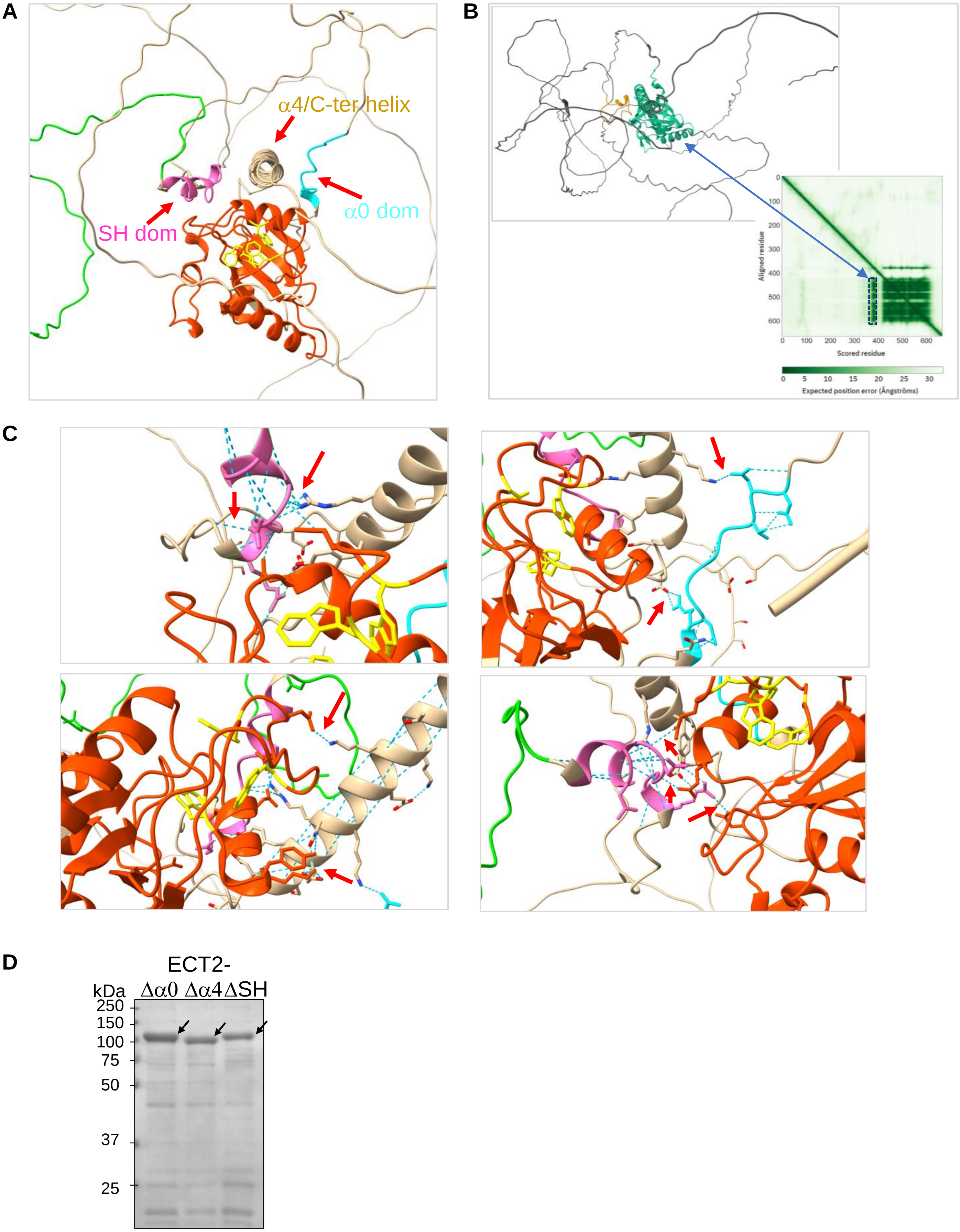

Supplemental Figure 5. Interactions between predicted folded domains of ECT2

A. Representation of the predicted 3D structure of ECT2 obtained from Alphafold using the Chimera software. The 303-375 domain is shown as green ribbon, the SH domain in magenta, the α0 3_{10} helix in blue, the α4/C-terminal helix in beige and the YTH domain in orange. The three tryptophans involved in m^6A recognition are shown in yellow.

B. Predicted aligned error (PAE) obtained from Alphafold. The PAE measures the confidence in the relative position of two residues within the predicted structure. The relative position of residues in the yellow square (aa 370-395, containing the SH domain, in orange in the upper left panel) are predicted with a high confidence when aligned with residues 420 to 616 (YTH domain with α0 helix and part of the α4 helix, in green in the upper left panel).

C. Zoom in of the predicted 3D structure of ECT2 showing the hydrogen bonds predicted between the SH domain (in magenta) and the α4/C-terminal helix (upper left panel), the α0 domain (in blue) and the α4/C-terminal helix (upper right panel), the α4/C-terminal helix and the YTH domain (left bottom panel). Hydrogen bonds are indicated by the arrows.

D. Coomassie stained-gel showing the purified recombinant His-SUMO tagged deletion proteins ECT2-Δα0, -Δα4 and -ΔSH.

Supplemental Figure 6

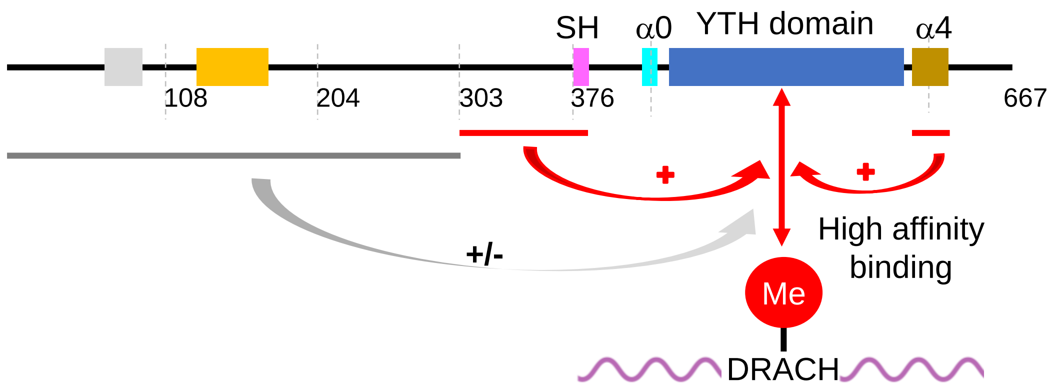

Supplemental Figure 6. Summary of the contributions of the regions and structural elements outside the YTH domain to ECT2 binding to m^{6}A *in vitro*. Schematic representation of the ECT2 full length protein, with the SLiM, YPQ-Rich, SH, α0, YTH and α4, represented with the same color code as previously. The amino acid positions of the successive N-terminal deletions are noted and marked with vertical dotted lines.

Supplemental Figure 7

DFB

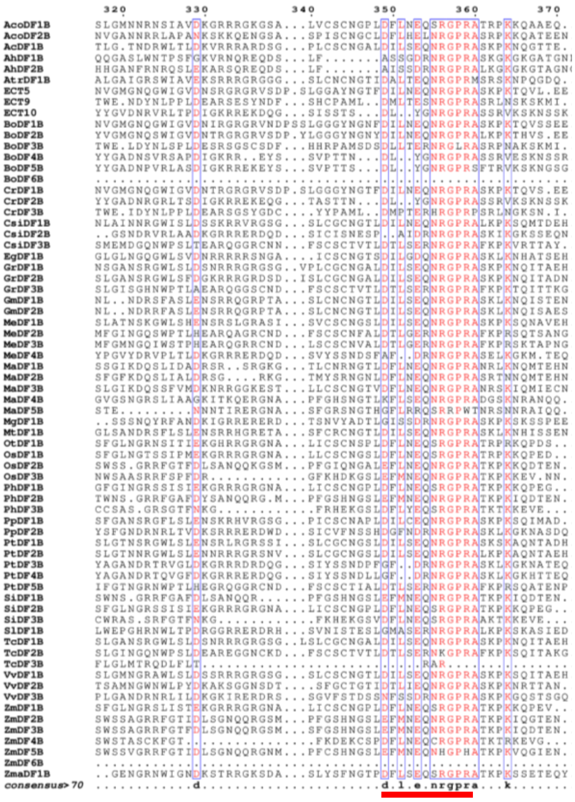

DFC

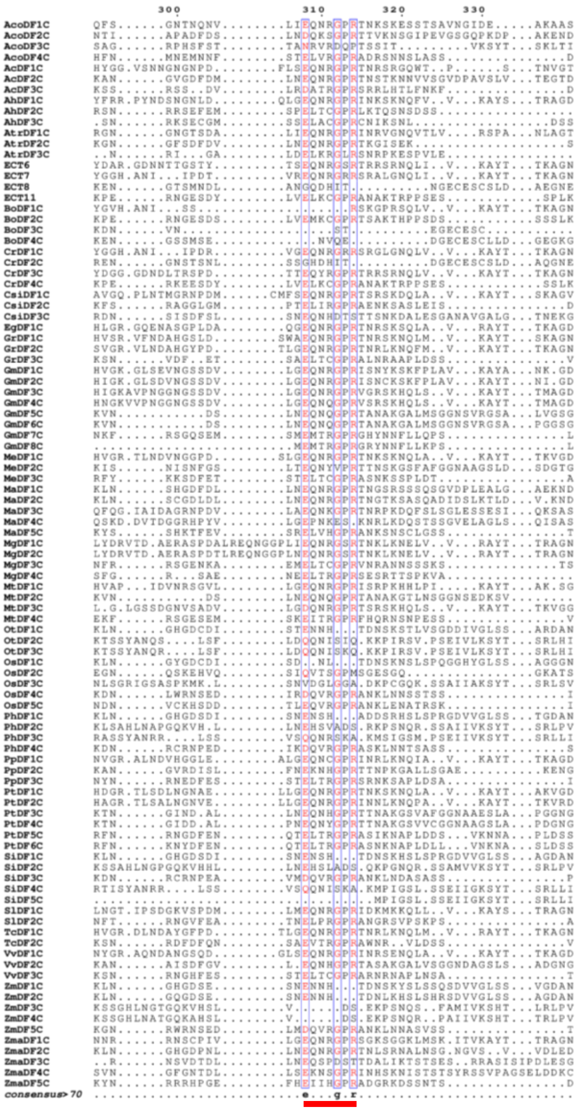

Supplemental Figure 7. Conservation of the SH domain in angiosperm YTHDF proteins. Alignments of DFB or DFC-type proteins (see sequences in the Suppl. Table S3, DFC proteins as defined in [5]) were done using the ESPript 3.0 software. The conserved regions corresponding to the ECT2 SH domain are underlined in red. Only the part of the sequence alignments surrounding the SH domain is shown.

Supplemental Figure 8

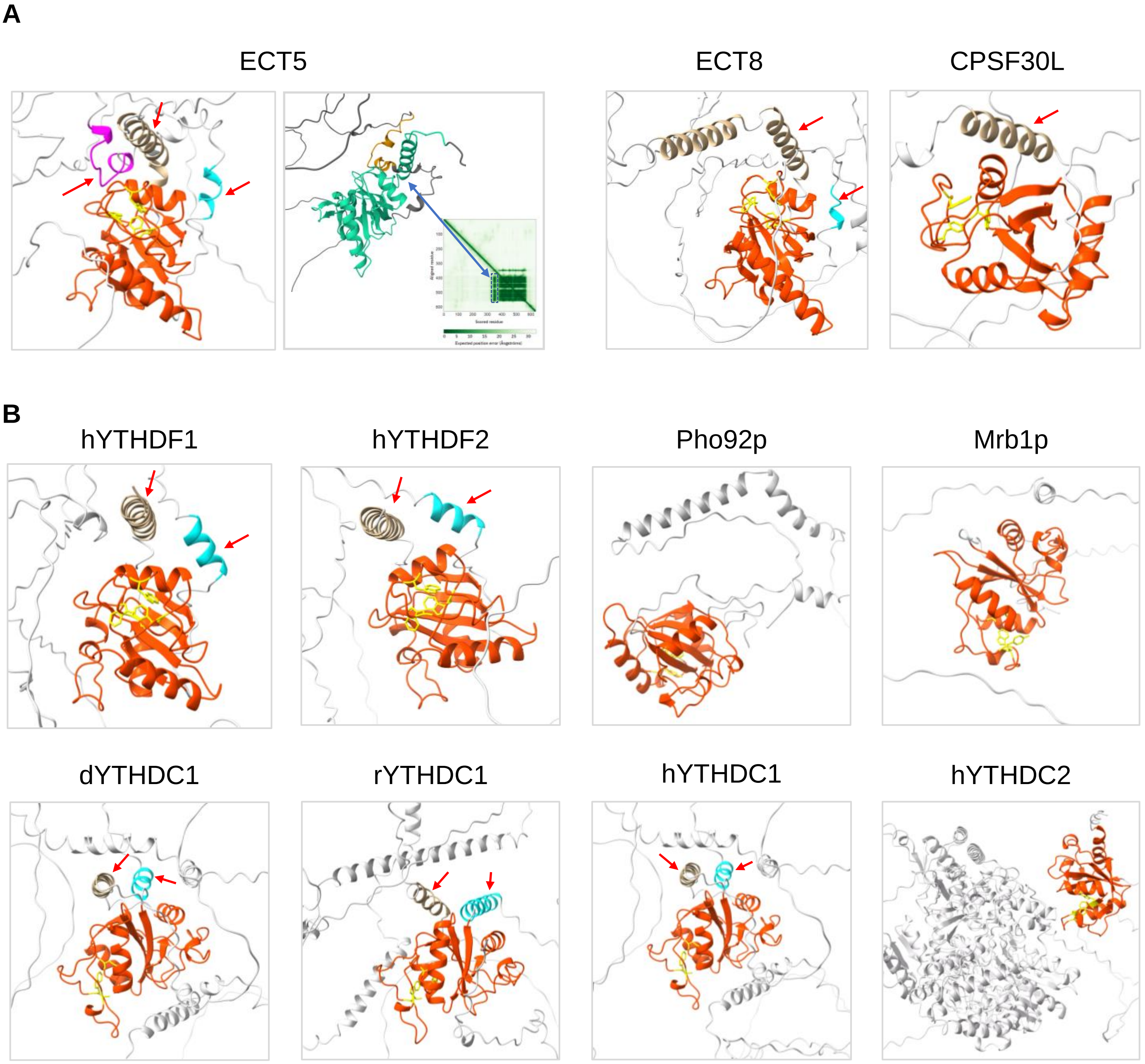

Supplemental Figure 8. Predicted 3D structures of YTH proteins from *Arabidopsis thaliana*, yeast, insect and mammals.

Alphafold-based 3D structure predictions of, in **A**, full-length Arabidopsis YTH proteins (ECT5 and ECT8 proteins respectively belonging to the DFB and DFC clades, and of one DC-type proteins, CPSF30-L); and in B, full-length proteins corresponding to previously biochemically characterized YTH domains from human DF proteins (hYTHDF1-2) and DC proteins (yeast Pho92p and Mrb1p, rat DC1 (rYTHDC1), Drosophila DC1 (dYTHDC1), human DC1 and 2 (hYTHDC1-2)). In A and B, the predicted 3D structures corresponding to the SH domain, α0 and α4 helices of ECT2 are shown respectively in magenta, blue and beige. They are indicated with the red arrow when structurally conserved compared to ECT2 3D predicted structure.
